# Quantification of training‐induced alterations in body composition via automated machine learning analysis of MRI images in the thigh region: A pilot study in young females

**DOI:** 10.14814/phy2.70187

**Published:** 2025-01-29

**Authors:** Saied Ramedani, Ebru Kelesoglu, Norman Stutzig, Hendrik Von Tengg‐Kobligk, Keivan Daneshvar Ghorbani, Tobias Siebert

**Affiliations:** ^1^ Graduate School of Cellular and Biomedical Sciences University of Bern Bern Switzerland; ^2^ Department of Diagnostic, Interventional and Pediatric Radiology Bern University Hospital, University of Bern Bern Switzerland; ^3^ Prokando GmbH Maybachstraße 27 Remseck am Neckar 71686 Germany; ^4^ Motion and Exercise Science University of Stuttgart Stuttgart Germany; ^5^ Stuttgart Center of Simulation Science University of Stuttgart Stuttgart Germany

**Keywords:** body composition, deep learning, machine learning, magnetic resonance imaging, musculoskeletal system, sports medicine

## Abstract

The maintenance of an appropriate ratio of body fat to muscle mass is essential for the preservation of health and performance, as excessive body fat is associated with an increased risk of various diseases. Accurate body composition assessment requires precise segmentation of structures. In this study we developed a novel automatic machine learning approach for volumetric segmentation and quantitative assessment of MRI volumes and investigated the efficacy of using a machine learning algorithm to assess muscle, subcutaneous adipose tissue (SAT), and bone volume of the thigh before and after a strength training. Eighteen healthy, young, female volunteers were randomly allocated to two groups: intervention group (IG) and control group (CG). The IG group followed an 8‐week strength endurance training plan that was conducted two times per week. Before and after the training, the subjects of both groups underwent MRI scanning. The evaluation of the image data was performed by a machine learning system which is based on a 3D U‐Net‐based Convolutional Neural Network. The volumes of muscle, bone, and SAT were each examined using a 2 (GROUP [IG vs. CG]) × 2 (TIME [pre‐intervention vs. post‐intervention]) analysis of variance (ANOVA) with repeated measures for the factor TIME. The results of the ANOVA demonstrate significant TIME × GROUP interaction effects for the muscle volume (*F*_1,16_ = 12.80, *p* = 0.003, *η*_*P*_^*2*^ = 0.44) with an increase of 2.93% in the IG group and no change in the CG (−0.62%, *p* = 0.893). There were no significant changes in bone or SAT volume between the groups. This study supports the use of artificial intelligence systems to analyze MRI images as a reliable tool for monitoring training responses on body composition.

## 1 INTRODUCTION

Clinical and research studies strongly recommend an appropriate ratio of body fat to muscle mass as an essential factor in maintaining health (Chumlea et al., 2002; Wells, 2005) and performance (Ackland et al., 2012). Several studies have shown that inappropriate amounts of fat can considerably raise the risk of many diseases. Particularly in the current era where obesity is a major contributor to the global burden of diseases (Finucane et al., 2011). The precise segmentation of different structures in the body is a very important task to investigate body composition.

The progressive development of imaging techniques such as computed tomography (CT), dual‐energy X‐ray absorptiometry (DEXA), and magnetic resonance imaging (MRI) offers the possibility to assess body composition involving whole‐body and/or regional muscle and fat mass (Franchi et al., 2018). MRI as an imaging method can assess the volume of body components (Haeufle et al., 2020; Hiepe et al., 2014). The main benefit of MRI in comparison to other techniques (e.g., CT scan) is the ability to precisely map the regional body composition without ionizing radiation (Kullberg et al., 2009). The latest developments in fat and water discrimination (e.g., Dixon sequence) using 3D multi‐echo gradients have further improved soft tissue contrast and the measurement accuracy of fat infiltration in skeletal muscle achievable by MRI (Ma, 2008). State‐of‐the‐art technologies allow the entire body to be scanned with an adequate resolution for body composition analysis in just less than 10 min (Kullberg et al., 2009). While MRI might be more expensive and less accessible compared to other methods for assessing body composition, it is important to note that its unique advantages and diagnostic value outweigh the cost and accessibility issues in many scientific studies. Furthermore, the use of MRI technology is becoming increasingly available and affordable in research settings, and advancements in imaging technology are making MRI more accessible to a wider range of research institutions and clinicians. In recent years, machine learning (ML) approaches and deep neural networks were developed to analyze acquired MRI images automatically (Liu et al., 2021; Ogier et al., 2021; Singhal et al., 2022). A system that fully automatically analyzes body composition will help evaluate the effectiveness of appropriate intervention programs.

It is known from the literature that strength training is an appropriate method for changing body composition (Ghasemikaram et al., 2021; Treuth et al., 1994). It was demonstrated that a 10 weeks strength training of elderly lead to an increase of muscle volume while intermuscular fat and SAT did not change (Hanson et al., 2009). However, other strength training studies found a decrease in fat mass (Karsten et al., 2021; Treuth et al., 1994).

Those studies are very important to evaluate appropriate training programs with the aim to change body composition and maintaining health. However, the traditional manual segmentation and evaluation of MRI data can be laborious, time‐consuming, and impractical for large‐scale studies. Thus, the integration of machine learning algorithms can enhance the accuracy and efficacy of training and rehabilitation monitoring processes for individuals, thereby facilitating more precise and personalized assessments of progress.

The aim of this study is to leverage our AI‐based image segmentation to validate a detailed analysis of muscle hypertrophy and changes in body composition following strength endurance training. By employing advanced machine learning techniques, we aim to provide a more accurate, efficient, and scalable method for assessing muscle, subcutaneous adipose tissue (SAT), and bone volume of the thigh before and after the intervention. It is hypothesized that strength training results in an increase in muscle volume and a decrease in SAT volume, while bone volume remains unchanged.

## 2 METHODS

### 2.1 Subjects

In order to increase the visibility of any potential impact on adipose tissue following the interventions, female participants were selected for this study. Eighteen healthy, young, female volunteers were recruited through social media. To meet the participating criteria, the subjects were restricted to low or no physical activity. This means that their physical activity should not exceed 1–2 days within a week. They were also required to have no injuries or physical limitations. They were randomly allocated to two groups: control group (CG) (*n* = 9; age: 26.1 ± 5.7; height: 167.3 ± 4.7; weight: 66.9 ± 10.1 kg), intervention group (IG) (*n* = 9; age: 27.3 ± 5.3; height: 164.7 ± 3.9 cm; weight: 63.4 ± 4.2 kg). The methods employed in this study were in accordance with the relevant guidelines and regulations, including the Declaration of Helsinki. The ethics committee of the University of Stuttgart (Az.21–041) gave its approval, and all participants signed a written informed consent.

### 2.2 Experimental design

In this study, each subject underwent two MRI measurements (pre‐intervention and post‐intervention). Between the two examinations, there was an interval of 8 weeks. During this time, the subjects who were in the IG had to carry out a training plan. The CG was instructed not to make any changes to their previous daily routine and explicitly not to do any sports with a high training load. Both the MRI scan and the training plan focused primarily on the thigh area. After both measurements, the MRI data was analyzed and evaluated by the ML‐system to measure muscle, SAT, and bone volumes.

**FIGURE 1:**
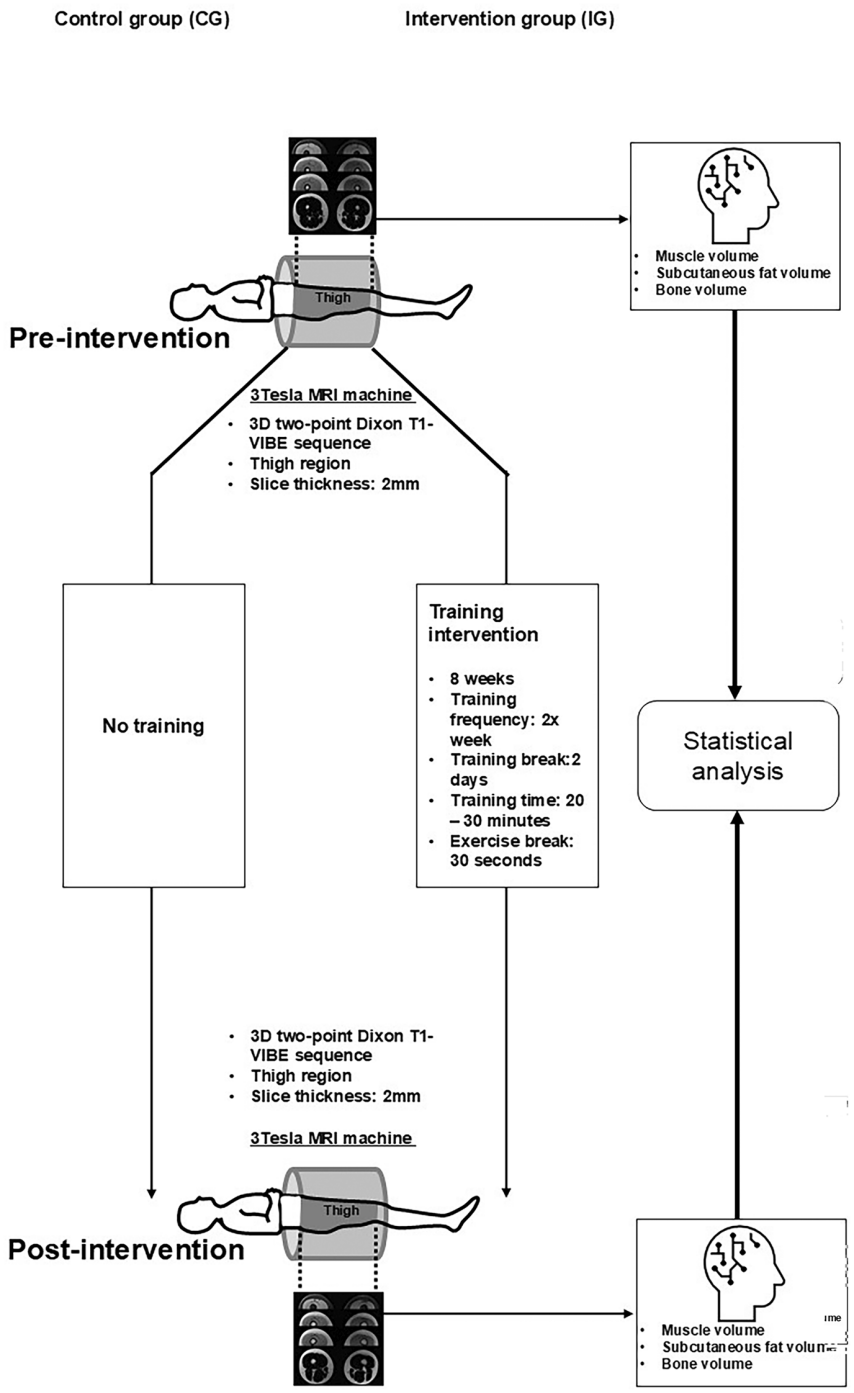
Experimental design of the study. A randomized controlled trial was conducted to examine the effects of strength endurance training on body composition in young, healthy women. The intervention group (IG) participated in an exercise plan for 8 weeks, twice a week, with a minimum of two full days without training between sessions), while the control group (CG) was instructed to maintain their usual lifestyle. Both groups underwent MRI measurement at two time points: before and after the study, with an eight‐week interval between measurements. MRI data from both groups were analyzed using a machine learning system trained to segment and quantify thigh muscle, SAT, and bone volumes. The volume measurements obtained were subjected to statistical analysis to detect significant differences between the two groups.

Figure 1 shows an overview of the experimental design of this study.

### 2.3 MRI acquisition

**FIGURE 2:**
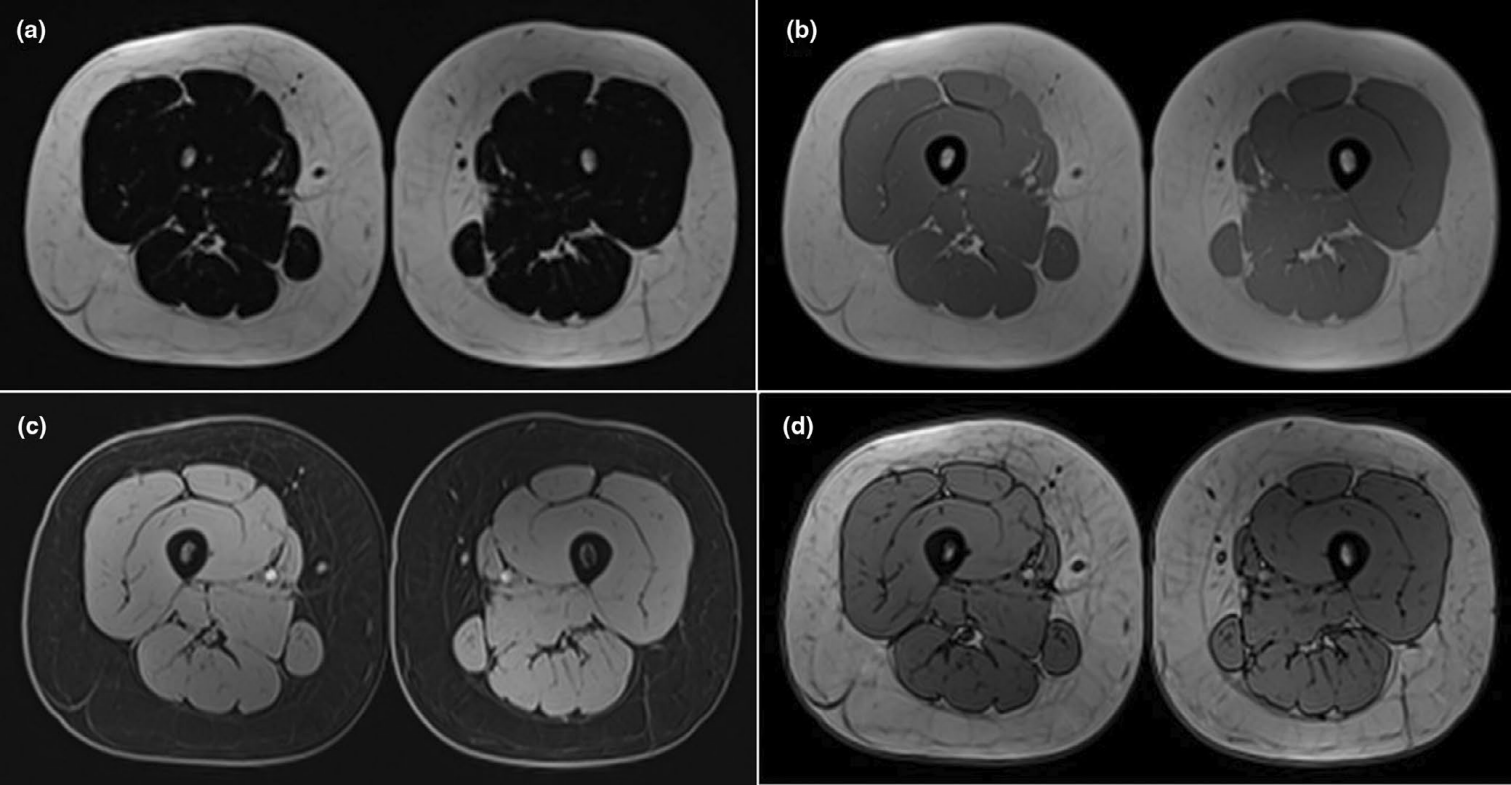
Four contrast images generated by Dixon technique in one measurement: (a) fat image in‐phase, (b) in‐phase, (c) water image, and (d) opposed‐phase.

3D MRI scans were acquired using the 3T MRI machine (Magnetom Lumina, Siemens Healthineers, Erlangen, Germany) with a 3D two‐point Dixon VIBE sequence for in‐phase, out‐of‐phase, water, and fat imaging (Figure 2) before and after the training period. Acquisition parameters were as follows: Percent Phase Field of View: 81.25, slice thickness: 2 mm, echo trains per slice: 2, repetition time (TR): 6 ms, echo time (TE): 1.23 ms, matrix: 286 × 352, flip angle: 6.5, slice over sampling: 75, Patient Position: FFS (Feet First‐Supine). To ensure that we examined the same muscle region at each time point of the MRI scan, we used a slice plane that was a fixed distance of 10 cm proximal to the lateral joint line of the knee joint in each subject. We also manually checked that every time we analyzed the same region in every subject. Specifically, 110 slices were analyzed for every subject, except for three subjects who had 96 slices in the pre‐ and post‐intervention due to differences in subject size. This approach allowed us to precisely compare changes in muscle size and structure over time, with confidence that we were analyzing the same region in each subject at each time point.

### 2.4 Interventions

As the study was conducted in times of the COVID‐19 pandemic, the access to gyms was limited. Therefore, a form of training was preferred that could be done at home or in a place of the participants' own choice and without additional weights. The 8‐week strength endurance training was carried out by the IG group only. Strength endurance training is characterized by >13 repetitions with weights less than 67% of the maximum strength value (1RM) (Baechle & Earle, 2008). Strength endurance training leads to an increase in muscle strength, muscle cross‐section (CSA), and a reduction in fat content (Fleck & Kraemer, 2004). The training was carried out twice a week, with a minimum of two full days without training between sessions, for 20–30 min, progressing through increasingly difficult exercises. To ensure proper execution of the training program, participants were provided with detailed instructions and videos demonstrating the correct form and technique for each exercise. To ensure compliance with the prescribed training program, the research team maintained regular contact with the participants and inspected their training sessions, although no formal documentation was required. The authors relied on oral communication with the participants to monitor and evaluate their training progress. Table S1 gives an overview about the exercises. The strength endurance training focused mainly on the hip and thigh muscles and consisted entirely of bodyweight exercises. No additional equipment, such as bands or dumbbells, was used. Each training session consisted of several (Finucane et al., 2011; Franchi et al., 2018; Hiepe et al., 2014) exercise series which were repeated three times (3 rounds). Rest between exercise series and rounds was 30 s and 3 min, respectively.

Due to the fact that the subjects were minimally active in sports, the eight‐week training plan was divided into three phases with increasing training effort: I. introductory week (week 1), II. difficulty level 1 (week 2–4), III. difficulty level 2 (week 5–8). In the introductory week, the test persons had time to become familiar with the exercises and to get to know the procedure and the execution of the individual exercises. For this phase, there was no time specified per exercise series, and the program began with exercises such as squats, lunges (for both legs), glute bridges, and wall sits. Instead, each participant was asked to learn proper and fluent movement execution (given by the video instruction). To increase the training effort in difficulty level 1, time under tension (TUT) was increased (Cintineo et al., 2018) by specifying a slow execution of the movement (5 s/ exercise repetition). Furthermore, the number of exercise repetitions was increased per week (Table S1) which results in an increased time per exercise series due to given slow movement velocity. In difficulty level 2, new and tougher exercises, such as squat‐jumps, reverse lunges, side lunges, and glute bridges with isometric holds have to be performed. Again, the number of exercise repetitions was increased per week.

### 2.5 Machine learning based analysis

The evaluation of the MRI image data utilized a novel machine learning system based on our previously published work, which integrates the four contrast images generated by the Dixon MRI (Ramedani et al., 2023). These images are crucial as they significantly enhance the signal‐to‐noise ratio, improve tissue contrast, and mitigate susceptibility artifacts, thereby providing more precise anatomical information. The development of the machine learning model in that study was conducted in accordance with established guidelines and regulations, including the Declaration of Helsinki. Data were obtained from Bern University Hospital (Inselspital) with explicit patient consent and ethical approval granted by the Ethics Commission of Canton Bern, Switzerland (Project‐ID: 2021–00846). Whole‐body MRI scans were acquired using a 3D two‐point Dixon VIBE sequence on a 3T Magnetom Vida Fit (Siemens Healthineers, Erlangen, Germany), yielding in‐phase, out‐of‐phase, water, and fat imaging data for analysis. The study cohort comprised 19 patients ranging from 18 to 90 years of age, ensuring comprehensive evaluation across diverse demographic groups. The system is designed to work with images of any size and dimension. During the pre‐processing stage, the images are divided into multiple sub‐volumes, allowing them to be effectively processed by the neural network. These sub‐volumes are created in an arbitrarily cube‐shaped format, enabling the network to comprehend and analyze complex image structures accurately. To expedite the processing, a novel approach was devised to partition MRI scans into smaller patches, containing a maximum amount of information, where only those patches comprising at least 5% foreground were selected for further analysis. For the training phase, ultimately, 75% of the selected sub‐volumes were designated as the training data, with the remaining sub‐volumes used for validation purposes.

The convolutional neural network (CNN) architecture of the system is inspired by the 3D U‐net by Kolařík et al. (Kolařík et al., 2019) which itself is based on the original U‐net (Ronneberger et al., 2015) implementation. The network was additionally improved and supplemented by adding more connections between the layers.

This enhanced design allows the network to effectively encode intricate features from input data. In the decoding phase, the network reconstructs images using these encoded features, maintaining high spatial resolution through bidirectional connections between encoding and decoding paths. This feature localization capability is pivotal for generating accurate images based on critical anatomical details captured in the MRI scans.

One of the techniques used to develop the network was the application of attention gates (Oktay et al., 2018). Attention gates are employed in the context of image segmentation to focus on relevant activations during CNN model training and minimize computational resources devoted to irrelevant activations, thus enhancing the network's generalization capability. U‐Net adopts skip connections during upsampling to overcome the problem of inaccurate spatial information. However, this leads to a proliferation of redundant low‐level feature extractions due to poor feature representation in the initial layers. Attention gates incorporated into the skip connections selectively suppress activations in irrelevant regions and reduce the transmission of redundant features. The attention mechanism assigns different weights to various sections of the image, amplifying areas of high relevance while minimizing those of low relevance. Moreover, due to these optimizations, the network is able to work with an extremely low amount of training data.

**FIGURE 3:**
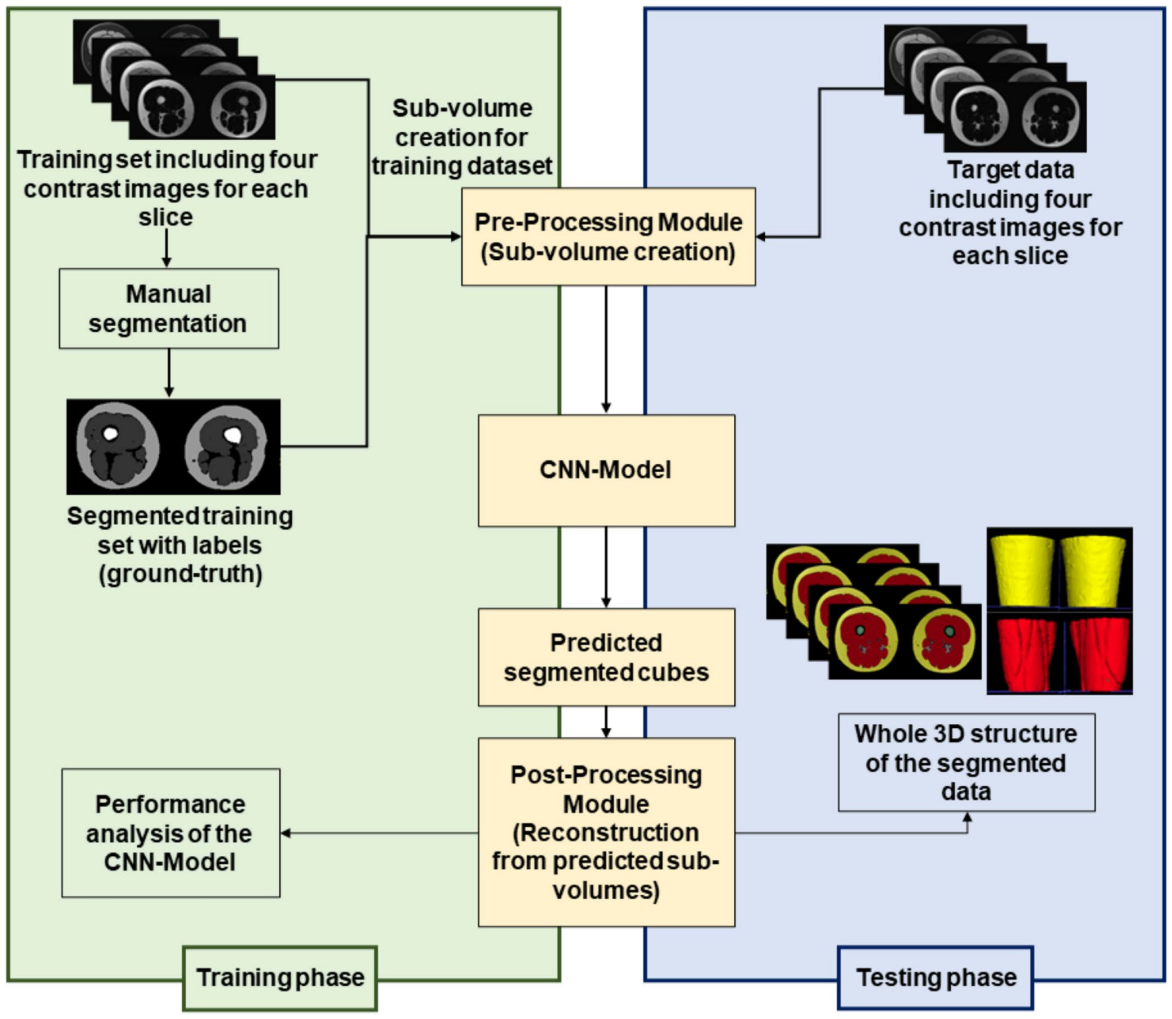
Flowchart of the ML‐based segmentation method. In the training phase, the CNN (Convolutional Neural Network) model is trained using a training set and its labels, which consist of whole‐body MRI images provided by University Hospital Bern. The pre‐processing module is utilized to create multiple cubes from these images and their associated labels for training. In the testing phase, the pre‐processing module is also used to create sub‐volumes from the unseen target data, which comes from the University of Stuttgart. The trained model then predicts the segmentation of these sub‐volumes. The post‐processing module reconstructs the segmented data into a 3D structure, representing the original form of the target data.

This model was subsequently employed for the purpose of voxel classification in the target images. Following the prediction process, individual blocks were reconstructed to the original form of the target data set. This reconstruction entailed assembling the predicted pieces into a coherent 3D structure, which served as the basis for volumetric calculations of the segmented structures. The calculated volumes of thigh muscle, SAT, and bone are used as input for the statistical analysis. The overall scheme of the ML‐based system is shown in Figure 3.

The machine learning system used in this study showed remarkable accuracy, with impressive Dice coefficients (DSC) for each segmented structure. For thigh muscle, our model achieved a DSC of 91.5%, while for subcutaneous adipose tissue (SAT) it achieved an even higher DSC of 96%. The model also achieved a DSC of 94% for bone segmentation. Gaj et al. (2023) scored a mean DSC of 93% when segmenting thigh muscle, while Kway et al. (2021) achieved a DSC of 94.3% when segmenting SAT. In addition, Almajalid et al. (2022) achieved a DSC of 95.2% for a similar segmentation task. These results highlight the excellent performance of our model, outperforming previous approaches in certain areas, and contribute to the increased credibility and accuracy of our volumetric measurements. These results provide a high level of confidence in the reliability and effectiveness of our machine learning model for segmentation.

### 2.6 Statistical analysis

For statistical interpretation, ordinary analyses such as means and standard errors (SE) were calculated. All data were tested for normal distribution using the Kolmogorov–Smirnov test and fulfilled the criteria. The volumes of muscle, bone, and SAT were analyzed using a 2 (GROUP [IG vs. CG]) × 2 (TIME [pre‐intervention vs. post‐intervention]) analysis of variance (ANOVA) with repeated measures for the factor time. The effect sizes were determined with the partial eta squared ($\eta_{P}^{2}$).

$$\eta_{P}^{2}=\frac{{\mathrm{SS}}_{\text{between}}}{{\mathrm{SS}}_{\text{between}}+{\mathrm{SS}}_{\text{error}}};$$

where ${\mathrm{SS}}_{\text{between}}$ = sums of squares of the interested effect. ${\mathrm{SS}}_{\text{error}}$ = sums of squares of the error term of the interested effect.

The effect sizes were classified as low ($\eta_{P}^{2}$ = 0.01), medium ($\eta_{P}^{2}$ = 0.06), and large ($\eta_{P}^{2}$ = 0.14) based on benchmarks provided by Cohen (Cohen, 2013). Significant main effects or interactions of the ANOVA were followed up using Tukey HSD tests. The significance level was set at *p* < 0.05. All analyses were performed using Statistica 14.0.0.15 software (TIBCO Software Inc., Palo Alto, California, USA).

## 3 RESULTS

### 3.1 Volume assessment

**FIGURE 4:**
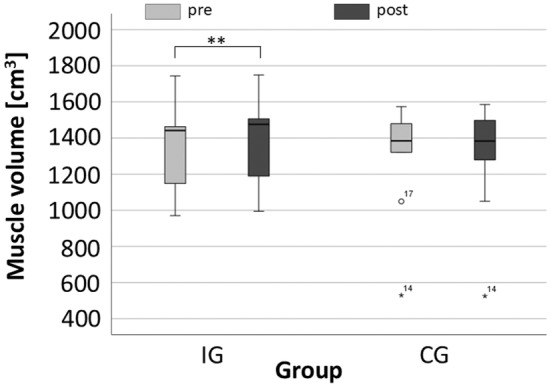
Mean and standard error of muscle volume for the intervention (IG) and control group (CG). The light gray bars represent the pre‐intervention and the dark gray bars the post‐intervention. The stars demonstrate high significant differences (*p* < 0.01). The box in the Box and Whisker plots represents the interquartile range (IQR), showing the middle 50% of the data (25th to 75th percentile), while the horizontal line inside the box represents the median (50th percentile). Whiskers extend to the minimum and maximum values, excluding outliers. Outliers are marked with circles (e.g., Subject ID 17 (see Table S2) in CG pre‐intervention) or stars (e.g., Subject ID 14 (see Table S2) in both pre‐ and post‐intervention of CG).

The results of the ANOVA demonstrate significant TIME × GROUP interaction effects for the muscle volume (*F*_1,16_ = 12.80, *p* = 0.003, *η*_*P*_^*2*^ = 0.44). Both the intervention group (IG) and control group (CG) consisted of 9 participants each. The muscle volume increased significantly in the IG group by 2.93% from the baseline value (*p* = 0.003). In contrast, the muscle volume of the CG did not change (−0.62%, *p* = 0.893) (Figure 4).

**FIGURE 5:**
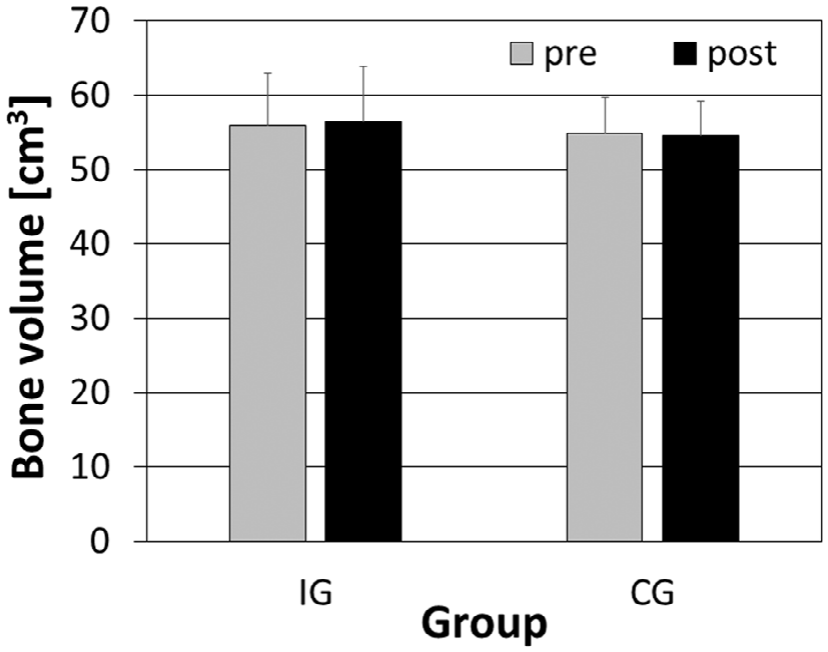
Mean and standard error of bone volume for the intervention (IG) and control group (CG). The black bars represent the post‐intervention, and the gray bars the pre‐intervention.

Furthermore, no differences were observed for bone volume (*F*_1,16_ = 2.64, *p* = 0.123, *η*_*P*_^*2*^ = 0.14) (Figure 5).

**FIGURE 6:**
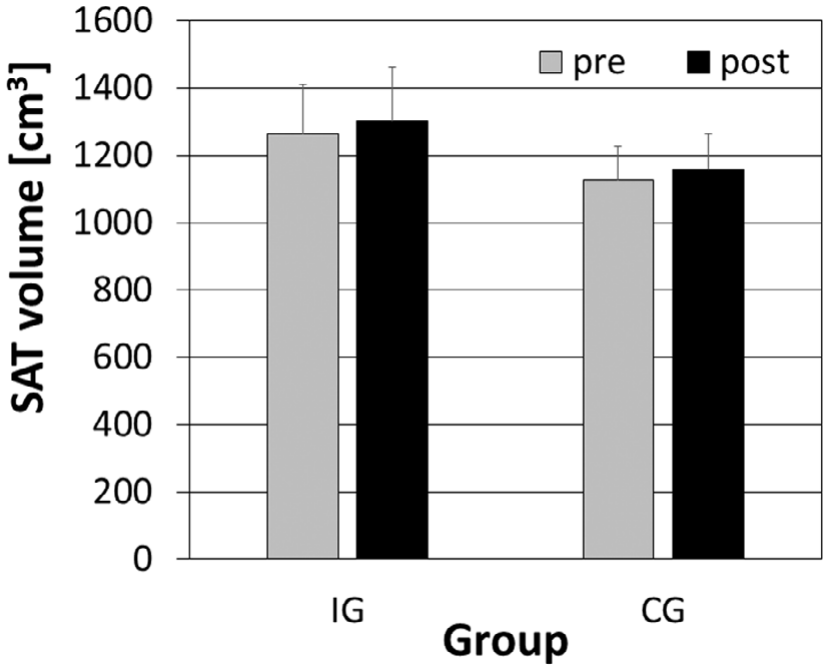
Mean and standard error of SAT volume for the intervention (IG) and control group (CG). The black bars represent the post‐intervention, and the gray bars the pre‐intervention.

We observed a significant difference for the factor TIME of the SAT volume (*F*_1,16_ = 5.58, *p* = 0.031, *η*_*P*_^*2*^ = 0.26). It increased from 1195 to 1229 cm^3^ by 2.8%. Conversely, there were no significant differences between the groups (*F*_1,16_ = 0.57, *p* = 0.459, *η*_*P*_^*2*^ = 0.03) and no interaction effects (*F*_1,16_ = 0.06, *p* = 0.815, *η*_*P*_^*2*^ = 0.04) (Figure 6).

Table S2 summarizes the measurement results of the volumes in the pre‐ and post‐intervention and provides details on the change in muscle, SAT, and bone volume.

## 4 DISCUSSION

This study reports significant thigh muscle hypertrophy in the IG group by 2.93% from baseline (*p* = 0.003) after 8 weeks of strength endurance training with a frequency of only two times per week. Training studies with comparable training programs demonstrated significant hypertrophic effects (Hanson et al., 2009; Treuth et al., 1994). Interestingly, the resistance load of the training program seems not to determine the increases of muscle volume (Schoenfeld et al., 2015). Mitchell et al. (Mitchell et al., 2012) conducted a training study and compared two different strength training programs: one group trained with resistance loads of 80% of 1 RM while another group trained with 30% of 1RM. Each group performed three sets of knee extensions to the point of fatigue. They observed increases in muscle volume for both groups of ca. 7% (Mitchell et al., 2012), which is higher than in the present study. However, Mitchell et al. (Mitchell et al., 2012) conducted the strength training program for 10 weeks with 3 training sessions per week, while we performed only 2 training sessions per week. Karsten et al. (Karsten et al., 2021) conducted a strength training program with 2 training sessions per week over 6 weeks and observed an increase of the vastus medialis thickness of 3.3% which is comparable to our study. It seems that the total training volume has a big impact on hypertrophic effects.

The effect of strength training on the SAT volume is inconsistent in the literature. We found no major differences between the groups when referring to SAT volume (Figure 6). Hanson et al. (Hanson et al., 2009) likewise found no difference in fat percentage after 10 weeks of strength training, while in Treuth's (Treuth et al., 1994) study, subcutaneous fat decreased by 9% after 16 weeks of training. Karsten et al. (Karsten et al., 2021) noticed a 1.5% reduction after 6 weeks of training.

The observed lack of significant change in SAT volume following the selected exercise program may suggest insufficient endurance training and restricted training sessions. This is consistent with the possibility that the training plan used in the study did not induce sufficient metabolic demand to elicit measurable changes in SAT volume. It is important to note that females generally have a higher SAT content compared to males. Therefore, the exclusive investigation of female participants in this study may have influenced the outcome, and it is unclear what the expected results would be in male participants. Furthermore, methodological limitations in determining the tissue proportions, for example, by manual segmentation of the tissue from MRI data might also influence the differences. To address this concern, we conducted a manual segmentation trial for two subjects (one from IG and one from CG) in two separate attempts by the same evaluator. The results of this analysis, summarized in Tables S3 and S4, revealed slight differences between the two trials, highlighting the inherent variability in manual segmentation methods. This exercise underscored the labor‐intensive nature of manual segmentation and reinforced the significant advantages of using machine learning for segmentation tasks. Here the ML‐based analysis offers a method to determine tissue proportions with high reproducibility.

In this study, our segmentation model demonstrated remarkable accuracy in segmenting 3D MR images when compared to the ground truth. The implementation of the machine learning technique for segmentation proved to be both fast and efficient, benefiting from the use of all four contrasts generated by the Dixon technique. To address concerns regarding the system intolerance while inputting all slices together into the network, we employed a sub‐volume technique, effectively reducing processing time and enhancing the overall training process. A significant strength of our approach lies in the incorporation of all four contrasts, as demonstrated by Yang et al. (Yang et al., 2016), which substantially improved tissue decomposition accuracy compared to using only one contrast image. This strategy proved particularly advantageous in segmenting small structures, as the additional contrast information compensated for noise and intensity non‐uniformity, rendering the algorithm more robust in classifying different tissues. Our use of machine learning for segmentation further alleviated challenges faced by clustering methods in cases with very thin subcutaneous fat or large intramuscular fat areas (Karlsson et al., 2015; Kemnitz et al., 2020), showcasing the superiority of our approach in accurately classifying different tissues.

In addition, this study provides insights regarding the advantages of machine learning over manual analysis or semi‐automatic approaches when evaluating the effects of the training plan on body composition. Compared to semi‐automatic methods that rely on manual annotation and interpolation (Azimbagirad et al., 2021), our machine learning framework offered significant advantages in terms of automation and scalability, making it well‐suited for large‐scale studies and clinical applications. By integrating automated methods, we reduced the potential for human error and variability inherent in manual analysis, ensuring more consistent and reliable results, particularly when applied to larger datasets or clinical studies.

The comparably fast analysis of changes in muscle and SAT volume is not only interesting for the individual evaluation of training plans and training methods, but it also has broader applications. The muscle volume data can, for example, also be used as input data for growth models of the skeletal muscles (Papenkort et al., 2021). Furthermore, the fast and correct recording of the 3D muscle and bone shapes enables an individualization of muscle skeletal models (Röhrle et al., 2017; Seydewitz et al., 2019) or human body models, which are increasingly used in biomechanics and vehicle development, for example, to examine and simulate human‐machine interaction during car driving or in crash test studies (Correia et al., 2021; Kempter et al., 2022; Putra et al., 2022).

## 5 CONCLUSION

This study proposes new approaches for the automatic evaluation of training‐induced alterations in body composition by using machine learning methods and shows significant advantages over manual analysis in terms of both time and quality. By leveraging these advancements, we have established an efficient, reliable, and precise method for monitoring volumetric changes resulting from training interventions. The implementation of fully automated methods not only enhances the efficiency of evaluation but also enables the seamless analysis of large‐scale studies. These advancements hold promise for transforming how exercise interventions are assessed and optimized in both research and clinical applications.

## AUTHOR CONTRIBUTIONS

This study was a collaborative effort with significant contributions from all authors. S.R. was responsible for software development, methodology, conceptualization, writing of the original draft, investigation, data curation, and visualization. E.K. contributed to the investigation and N.S. to the validation, formal analysis, and visualization. H.T.K. contributed to the review and editing, resources, and funding acquisition. K.D.G. provided supervision, review and editing, project administration, and funding acquisition. T.S. provided supervision, conceptualization, writing, review and editing, project administration, and funding acquisition. All authors reviewed and approved the final version of the manuscript, ensuring the integrity, and accuracy of the study.

## FUNDING INFORMATION

This study was financially supported by Prokando GmbH and partially funded by Deutsche Forschungsgemeinschaft (DFG, German Research Foundation) under Germany's Excellence Strategy – EXC 2075–390740016.

## CONFLICT OF INTEREST STATEMENT

The authors declare that the research was conducted in the absence of any commercial or financial relationships that could be construed as a potential conflict of interest.

## ETHICS STATEMENT

This study was conducted in compliance with the ethical standards set forth by the Ethics Committee of the University of Stuttgart, with approval granted under the reference number Az.21–041. Additionally, retrospective data for the purpose of training a machine learning model, were obtained from the Canton of Bern, Switzerland, with ethical approval obtained under Project‐ID: 2021–00846. The procedures performed in this study were in accordance with the relevant guidelines and regulations, which include the Declaration of Helsinki.

## Supporting information


Table S1.

## Data Availability

Due to the sensitive nature of the MRI data and the proprietary nature of the machine learning codes, these materials cannot be made publicly available. However, the processed data and analysis results can be made available from the corresponding author upon request.
