# Supplementary material for: Quantification of training‐induced alterations in body composition via automated machine learning analysis of MRI images in the thigh region: A pilot study in young females
Source: Physiol Rep. 2025 Jan 29;13(3):e70187. doi: 10.14814/phy2.70187 (PMC11776390; doi:10.14814/phy2.70187)
Supplement: Supplementary file 1 — Table S1. [file PHY2-13-e70187-s001.docx]

Supplementary Material

Supplementary Table S.1: An overview of the exercises and repetitions in: I. introductory week (week 1) II. Difficulty level 1 (week 2 to 5) III. Difficulty level 2 (week 5 to 8).

|  | **week** | **time per exercise series** | **Exercise repetitions per round (total 3 rounds)** |
| --- | --- | --- | --- |
| introductory week  (normal movement velocity: 2-4s/exercise) | 1 | - | 20 squats  12 lunges (left leg)  12 lunges (right leg)  15 glute bridge  1 wall-sit |
| difficulty level 1  -increased time under tension (slow movement velocity: 5s/exercise) | 2 | 30 | 6 squats  6 lunges (left leg)  6 lunges (right leg)  6 glute bridge  1 wall-sit |
|  | 3 | 40 | 8 squats  8 lunges (left leg)  8 lunges (right leg)  8 glute bridge  1 wall-sit |
|  | 4 | 50 | 10 squats  10 lunges (left leg)  10 lunges (right leg)  10 glute bridge  1 wall-sit |
| difficulty level 2  - new, tougher exercises  - increased time under tension (slow movement velocity: 5s/exercise) | 5 | 30 | 6 squat-jumps  6 reverse lunges (left leg)  6 reverse lunges (right leg)  6 side lunges (left leg)  6 side lunges (right leg)  1 glute bridge with isometric hold |
|  | 6 | 40 | 8 squat-jumps  8 reverse lunges (left leg)  8 reverse lunges (right leg)  8 side lunges (left leg)  8 side lunges (right leg)  1 glute bridge with isometric hold |
|  | 7 | 50 | 10 squat-jumps  10 reverse lunges (left leg)  10 reverse lunges (right leg)  10 side lunges (left leg)  10 side lunges (right leg)  1 glute bridge with isometric hold |
|  | 8 | 60 | 12 squat-jumps  12 reverse lunges (left leg)  12 reverse lunges (right leg)  12 side lunges (left leg)  12 side lunges (right leg)  1 glute bridge with isometric hold |

Supplementary Table S.2: Measurements of the volumes for muscle, fat and bone in the pre- and post-test. SD: Standard Deviation.

| **Subjects** | | **Pre- intervention** | | | **Post- intervention** | | | **Change (%)** | | |
| --- | --- | --- | --- | --- | --- | --- | --- | --- | --- | --- |
| **Subject ID** | **Group** | **Muscle volume (cm³)** | **Fat volume (cm³)** | **Bone volume (cm³)** | **Muscle volume (cm³)** | **Fat volume (cm³)** | **Bone volume (cm³)** | **Muscle volume** | **Fat volume** | **Bone volume** |
| **01** | **IG** | 1357 | 1528 | 37.61 | 1419 | 1590 | 37.9 | 4.4% | 3.9% | 0.8% |
| **02** | **IG** | 970 | 644.9 | 79.01 | 1018 | 643.5 | 80.49 | 4.7% | -0.2% | 1.8% |
| **03** | **IG** | 1445 | 1070 | 51.44 | 1476 | 1016 | 51.88 | 2.1% | -5.3% | 0.8% |
| **04** | **IG** | 1744 | 1701 | 32.29 | 1749 | 1696 | 33.03 | 0.3% | -0.3% | 2.2% |
| **05** | **IG** | 1442 | 933.1 | 51.22 | 1491 | 954.7 | 51.5 | 3.3% | 2.3% | 0.5% |
| **06** | **IG** | 1148 | 1915 | 43.68 | 1189 | 2005 | 42.28 | 3.4% | 4.5% | -3.3% |
| **07** | **IG** | 1553 | 1626 | 47.75 | 1638 | 1836 | 46.36 | 5.2% | 11.4% | -3.0% |
| **08** | **IG** | 993 | 1042 | 98.64 | 994.1 | 1054 | 101.7 | 0.1% | 1.1% | 3.0% |
| **09** | **IG** | 1463 | 909.7 | 61.77 | 1506 | 911.7 | 63.44 | 2.9% | 0.2% | 2.6% |
| **10** | **CG** | 1562 | 1055 | 42.92 | 1586 | 1076 | 43.59 | 1.5% | 2.0% | 1.5% |
| **11** | **CG** | 1479 | 1267 | 55.4 | 1498 | 1353 | 55.47 | 1.3% | 6.4% | 0.1% |
| **12** | **CG** | 1378 | 1448 | 44.1 | 1386 | 1550 | 44.53 | 0.6% | 6.6% | 1.0% |
| **13** | **CG** | 1384 | 1203 | 40.94 | 1383 | 1199 | 39.19 | -0.1% | -0.3% | -4.5% |
| **14** | **CG** | 529.2 | 932.6 | 83.42 | 524.8 | 911.9 | 82.89 | -0.8% | -2.3% | -0.6% |
| **15** | **CG** | 1407 | 1185 | 45.45 | 1339 | 1211 | 45.7 | -5.1% | 2.1% | 0.5% |
| **16** | **CG** | 1574 | 1567 | 50.98 | 1578 | 1570 | 50.84 | 0.3% | 0.2% | -0.3% |
| **17** | **CG** | 1049 | 922.9 | 61.26 | 1050 | 955.4 | 59.7 | 0.1% | 3.4% | -2.6% |
| **18** | **CG** | 1321 | 560.8 | 69.76 | 1279 | 590.5 | 69.42 | -3.3% | 5.0% | -0.5% |
| **mean** | **IG** | 1346.1 | 1263.3 | 55.9 | 1386.7 | 1300.8 | 56.5 | 2.9% | 2.0% | 0.6% |
| **SD** | **IG** | 244.8 | 411.1 | 19.8 | 248.9 | 455.5 | 20.9 | 1.7% | 4.3% | 2.2% |
| **mean** | **CG** | 1298.1 | 1126.8 | 54.9 | 1291.5 | 1157.4 | 54.6 | -0.62% | 2.56% | -0.59% |
| **SD** | **CG** | 308.7 | 284.9 | 13.5 | 312.2 | 297.5 | 13.3 | 2.06% | 2.89% | 1.76% |
|  |  |  |  |  |  |  |  |  |  |  |

|  |
| --- |

Supplementary Table S.3: Differences in muscle, fat and bone volume in two attempts (1, 2) of manual analysis by one evaluator for two subjects (03 and 15). Note that manual analysis of the MRI scans is a time-intensive process, taking several hours per subject for an evaluator.

| **Subjects** | |  | **Pre- intervention** | | | **Post- intervention** | | |
| --- | --- | --- | --- | --- | --- | --- | --- | --- |
| **Subject ID** | **Group** | **Attempt** | **Muscle volume (cm³)** | **Fat volume (cm³)** | **Bone volume (cm³)** | **Muscle volume (cm³)** | **Fat volume (cm³)** | **Bone volume (cm³)** |
| **03** | **IG** | 1 | 1382 | 1036 | 33.89 | 1565 | 910 | 31.06 |
| **03** | **IG** | 2 | 1584 | 1094 | 31.68 | 1681 | 1013 | 28.33 |
| **Difference* (%)** | **IG** | - | 14.6% | 5.6% | 6.5% | 7.4% | 11.3% | 8.8% |
| **15** | **CG** | 1 | 1440 | 1206 | 27.49 | 1424 | 1191 | 31.76 |
| **15** | **CG** | 2 | 1323 | 995.7 | 32.45 | 1251 | 1043 | 32.76 |
| **Difference* (%)** | **CG** | - | 8.1% | 17.4% | 18.0% | 12.1% | 12.4% | 3.1% |
| *** Difference: percentage of the volume difference absolute value** | | | | | | | | |

Supplementary Table S.4: Mean value of muscle, fat and bone volume in two attempts (1, 2) of manual analysis by one evaluator for two subjects (03 and 15).

| **Subjects** | |  | **Pre-intervention** | | | **Post- intervention** | | | **Change (%)** | | |
| --- | --- | --- | --- | --- | --- | --- | --- | --- | --- | --- | --- |
| **Subject ID** | **Group** | **Attempt** | **Muscle volume (cm³)** | **Fat**  **volume (cm³)** | **Bone volume (cm³)** | **Muscle volume (cm³)** | **Fat volume (cm³)** | **Bone volume (cm³)** | **Muscle volume** | **Fat volume** | **Bone volume** |
| **03** | **IG** | 1 | 1382 | 1036 | 33.89 | 1565 | 910 | 31.06 | 11.69% | -13.85% | -9.11% |
| **03** | **IG** | 2 | 1584 | 1094 | 31.68 | 1681 | 1013 | 28.33 | 5.77% | -8.00% | -11.82% |
| **Mean** | **IG** | - | 1483 | 1065 | 33 | 1623 | 962 | 30 | 8.7% | -10.9% | -10.5% |
| **15** | **CG** | 1 | 1440 | 1206 | 27.49 | 1424 | 1191 | 31.76 | -1.12% | -1.26% | 13.44% |
| **15** | **CG** | 2 | 1323 | 995.7 | 32.45 | 1251 | 1043 | 32.76 | -5.76% | 4.53% | 0.95% |
| **Mean** | **CG** | - | 1381.5 | 1100.85 | 29.97 | 1337.5 | 1117 | 32.26 | -3.4% | 1.6% | 7.2% |
|  |  |  |  |  |  |  |  |  |  |  |  |
